# Soil organic carbon, extracellular polymeric substances (EPS), and soil structural stability as affected by previous and current land-use

**DOI:** 10.1016/j.geoderma.2019.114143

**Published:** 2020-04-01

**Authors:** M. Redmile-Gordon, A.S. Gregory, R.P. White, C.W. Watts

**Affiliations:** a Environmental Horticulture Department, Royal Horticultural Society, Wisley, GU23 6QB, United Kingdom; b Sustainable Agriculture Sciences Department, Rothamsted Research, Harpenden, Hertfordshire, AL5 2JQ, United Kingdom; c Computational and Analytical Sciences Department, Rothamsted Research, Harpenden, Hertfordshire, AL5 2JQ, United Kingdom

**Keywords:** Transient binding agents, Microbial exudates, Protein matrix bonding, Exopolysaccharide, Water stable aggregate (MWD), Cation exchange resin (CER)

## Abstract

While soil microbial ecology, soil organic carbon (SOC) and soil physical quality are widely understood to be interrelated — the underlying drivers of emergent properties, from land management to biochemistry, are hotly debated. Biological binding agents, microbial exudates, or ‘extracellular polymeric substances’ (EPS) in soil are now receiving increased attention due to several of the existing methodological challenges having been overcome. We applied a recently developed approach to quantify soil EPS, as extracellular protein and extracellular polysaccharide, on the well-characterised soils of the Highfield Experiment, Rothamsted Research, UK. Our aim was to investigate the links between agricultural land use, SOC, transient binding agents known as EPS, and their impacts on soil physical quality (given by mean weight diameter of water stable aggregates; MWD). We compared the legacy effects from long-term previous land-uses (unfertilised grassland, fertilised arable, and fallow) which were established > 50 years prior to investigation, crossed with the same current land-uses established for a duration of only 2.5 years prior to sampling. Continuously fallow and grassland soils represented the poorest and greatest states of structural integrity, respectively. Total SOC and N were found to be affected by both previous and current land-uses, while extractable EPS and MWD were driven primarily by the current land-use. Land-use change between these two extremes (fallow → grass; grass → fallow) resulted in smaller SOC differences (64% increase or 37% loss) compared to MWD (125% increase or 78% loss). SOC concentration correlated well to MWD (adjusted *R*^2^ = 0.72) but the greater SOC content from previous grassland was not found to contribute directly to the current stability (p < 0.05). Our work thus supports the view that certain distinct components of SOC, rather than the total pool, have disproportionately important effects on a soil’s structural stability. EPS-protein was more closely related to aggregate stability than EPS-polysaccharide (*p* values of 0.002 and 0.027, respectively), and ranking soils with the 5 greatest concentrations of EPS-protein to their corresponding orders of stability (MWD) resulted in a perfect match. We confirmed that both EPS-protein and EPS-polysaccharide were transient fractions: supporting the founding models for aggregate formation. We suggest that management of transient binding agents such as EPS —as opposed to simply increasing the total SOC content— may be a more feasible strategy to improve soil structural integrity and help achieve environmental objectives.

## Highlights

• 2.5 years of current land-use influenced structural stability far more than the preceding 50 years.
• SOC provided a good R^2^ (0.72) but failed to predict the stability sequence of the more stable soils.
• EPS were transient binding agents: affected by the current land-use, but not preceding ones.
• Among EPS constituents, EPS-protein appeared to be most related to soil structural integrity.
• Stability gains from current land-uses were enhanced where preceding ones included plant-cover.

## 1 Introduction

### 1.1 Land use, total SOC and soil structural stability

It is well established that surface vegetation, as controlled by how land is managed, has profound interactions with the underlying soil. In this way, land-use affects many of a soil’s fundamental properties including soil organic matter, and its primary constituent: soil organic C (SOC; Jobbágy and Jackson, 2000). Managements that increase SOC in loamy soils enable the development of microporous networks, which are more conducive to the transmission of nutrients, water and air (Bacq-Labreuil et al., 2018, Guo et al., 2020). Without stability in soil structure, these networks and aggregates collapse: restricting porosity, connectivity, nutrient transport, infiltration and root penetration. Soils under arable crops are subjected to annual cultivation and most of the above-ground biomass is harvested, compared to soils under permanent vegetative cover, which have no such disturbance and benefit from increased perennial inputs of C (Chapman et al., 2013, Wiesmeier et al., 2012). It follows that a change in land use alters the balance between inputs and outputs and SOC adjusts to a new equilibrium accordingly (Gregory et al., 2016, Johnston et al., 2009). Soil typically loses SOC faster than it is gained (Johnston et al., 2009, Poeplau et al., 2011, Jones et al., 2016) and so international efforts to increase SOC, such as the ‘4-per-mille’ initiative (Chabbi et al., 2017) are challenged by high frequencies in land-use change.

Alternative policies to increasing SOC via direct inputs have been called for which focus more on improving soil physical quality (Poulton et al., 2018) and understanding microbial processes contributing to C storage (Liang, et al., 2017). The water-stability and size distribution of soil aggregates, as described by their mean weight diameter (MWD) reflects the stability against physical stresses typically imposed *in-situ* (Le Bissonnais, 1996). Accordingly, MWD is commonly used to build an index of soil health and/or quality. Regression models have gone as far as to explain aggregate stability by simple SOC content (e.g. Kemper and Koch, 1966, Six et al., 2002, Soinne et al., 2016) but the relationships between SOC and aggregate stability are not straightforward (Denef and Six, 2005).

### 1.2 Organic binding agents

Both the quality and location of SOC, or more specifically ‘binding agents’ at the microscale are understood to be important factors determining the overall stability of a soil (Denef et al., 2007, Jensen et al., 2019). The seminal work of Tisdall and Oades (1982) was perhaps the early thesis on this. They proposed an aggregate hierarchy where smaller microaggregates, held together by *persistent* binding agents (organo-mineral associations), or *transient* binding agents (microbial exopolysaccharides) combine within macroaggregates and are stabilised by *temporary* binding agents such as roots and fungal hyphae. Whilst persistent agents were thought to be largely regulated by soil mineralogy and particle size, both the temporary and transient agents were proposed to be controlled by land-use (Tisdall and Oades, 1982). Golchin et al. (1994) further developed the theory to include microbially-dependent cycles of aggregate formation and dispersal, commencing with organic matter inputs from plant cover which stimulate microbial production of transient binding agents. This in turn facilitated the adsorption of aligned clay particles, by electrostatic attraction, forming central units of new stable aggregates. When readily-available C became depleted, their model predicted binding agents would no longer be produced and the aggregates would crumble when subjected to physical stress. Soil biology has since been shown to be a major contributor to soil aggregate stability (Watts et al., 2001, Watts et al., 2005, Jensen et al., 2019), which is highly responsive to management (Kabir, 2005) and especially C inputs (Hirsch et al., 2017).

### 1.3 Extracellular polymeric substances (EPS)

The production of exudates as biological binding agents is a salient feature of individual microorganisms attached to their physical habitat or co-habiting in groups called biofilms (Flemming and Wingender, 2010). This exuded matrix is composed of biopolymers more widely referred to as *extracellular polymeric substances* or ‘EPS’, a term we use hereafter. EPS is thought to be composed primarily of polysaccharides but proteins are a crucial functional component (Flemming et al., 2007). Evidence from water sciences shows that protein-rich EPS plays a central role in adhesion and stabilisation of microbial aggregates (Guo et al., 2016). Indeed, *ex-situ* additions of bacteria selected for their propensity to exude proteinaceous EPS (e.g. *Pseudomonas* spp.) have been shown to aggregate soil particles in solution (Caesar-Tonthat et al., 2014, Krause et al., 2019). While extracellular polysaccharides have long been assumed to be important for soil structure, studies of EPS in soil have been held back by methodological challenges. For example, in the unique study of Tang et al., (2011) the addition of selective fungicides and bactericides were both shown to decrease soil aggregate stability but the methods used to measure extracellular polysaccharides failed to uncover any statistically significant link. Redmile-Gordon et al. (2014) investigated the ‘hot sulphuric acid’ method used in the study of Tang et al. (2011) and found that the method also co-extracted large amounts of intracellular biomass, and nonspecific extracellular SOC – likely confounding all three classes of biological binding agent. Other investigators have attempted to use hot water as an extractant but conceded that hot water was also not selective for extracellular polysaccharides (Marchus et al., 2018).

Related work surrounds the extraction of proteinaceous exudates of arbuscular mychorrhizal fungi, named glomalin, or glomalin-related soil proteins (GRSP), which are believed to impart aggregate stability and C storage (Wright and Upadhyaya, 1996, Bach et al., 2010). However, the autoclaving step precludes any claim that these extracts represent extracellular material. Furthermore, extracts of GRSP were shown to contain more proteins of bacterial than fungal origin (Gillespie et al., 2011). Redmile-Gordon et al. (2013) proposed that it might be more practical to measure changes in pools of categorically *extracellular* biopolymers as a biophysically distinct product of the microbial biomass, rather than attempting to ascribe taxonomic origin to materials that could be either intracellular or extracellular. The present lack of studies on EPS, linking soil structural stability, or the effects of land-use change represents an opportunity for researchers to advance our understanding of sustainable soil management in this rapidly expanding area (Costa et al., 2018, Liang et al., 2017).

### 1.4 Methodological developments for extracting EPS

The qualitative challenges described above suggest a priority for methods that do not co-extract large amounts of ‘non-target’ organic matter (intracellular or extracellular). The only method reported to meet these criteria to date is the ‘cation exchange resin’ (CER) method initially developed by Frolund et al. (1996) and adapted for application in soil by Redmile-Gordon et al. (2014). In the method for soils, a dilute divalent metal salt (CaCl_2_) is used to remove the easily soluble organic fraction. This fraction is highly variable in soil depending on rainfall and moisture content immediately prior to sampling. It is important that the preliminary extractant is not monovalent to maintain EPS stability prior to subsequent removal using CER. The resin extraction step works by reducing binding between negatively charged moieties of the polymeric matrix that are otherwise bridged by divalent cations and releases shorter chains into the extraction buffer (Sheng et al., 2010). This same method was also found to be suitable for EPS in soils dominated by trivalent metal cations (Wang et al., 2019).

The extracellular specificity of the soil EPS extraction method was confirmed using measurements of soil microbial adenosine 5′-triphosphate (ATP; Redmile-Gordon et al., 2011, Redmile-Gordon et al., 2014) and the findings concurred with investigators in aquatic sciences (e.g. Takahashi et al., 2009, Ge et al., 2010) that cell-lysis during CER extraction was minimal. To address concerns regarding extracellular contamination, Redmile-Gordon et al. (2015) traced stable isotopes of N through the microbial biomass, showing that CER preferentially extracted synthesised EPS over extracellular SOC of more dubious origin. Colorimetric measurements of EPS protein and polysaccharide in the soil extracts were also corroborated by GC–MS measurements of volatile derivatives, adding support to the more affordable colorimetric determination methods that are normally applied.

### 1.5 Aims and objectives

Our objectives were to investigate the effect of land use or management changes on soil EPS at the field scale and relate these findings to concomitant changes in soil aggregate stability as a fundamental soil physical property. Importantly, the combinations of ‘previous’ and ‘current’ land-uses in this long-term experiment would provide a contrast in the proportion of transient binding agents relative to the total organic matter pool: with ‘previous’ and ‘current’ each representing a deficit, and abundance, of land-use-driven binding agents, respectively. Accordingly, we tested three hypotheses: (1) The mixed ley/grass land-use would support the greatest production of microbial EPS, followed by arable, and fallow managements, respectively. (2) EPS concentrations would be more affected by short-term application of the most conducive management than by ‘legacy effects’ from previous managements – even when that previous management had been applied continuously for a duration of over 50 years. (3) These same EPS concentrations would be significantly correlated to an indicator of soil physical quality i.e. aggregate stability.

## 2 Material and methods

### 2.1 Experimental site

**Fig. 1:**
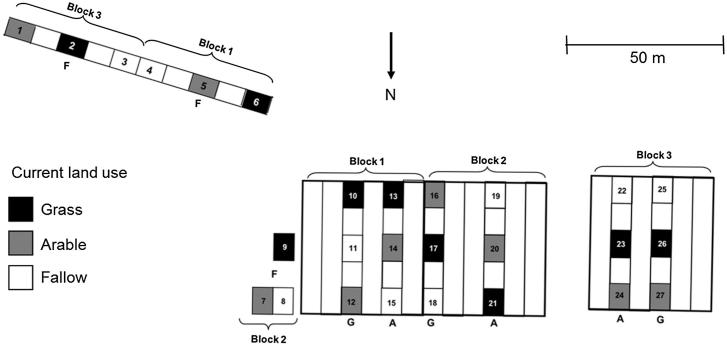
The Highfield experiment with previous grass (G), arable (A) (both since 1949, in 50 × 7 m plots) and bare fallow (F) (since 1959, in adjacent land totalling *c.* 1400 m^2^) land uses, with superimposed current grass (black), arable (grey) and fallow (white) land uses. The experimental design comprises 9 treatments triplicated in 27 split-plots (3 previous × 3 current land uses × 3 field replicates). Plot numbers are given.

This study was conducted on the Highfield experiment at Rothamsted Research, UK (Fig. 1; global coordinates 51.804, –0.363) where contrasting land-uses were established previously (duration >50 years) and recently (2.5 years prior to sampling). There is an annual mean temperature (1992–2014) and rainfall (1981–2010) of 10.2 °C and 718 mm, respectively (Scott et al., 2014). The soil is classified as a stagnogleyic paleo-argillic brown earth (Batcombe series), equivalent to a Chromic Luvisol (IUSS Working Group WRB, 2015) with silty clay loam texture (Avery and Catt, 1995). Mineralogy is dominated by expanding 2:1 clays (randomly interstratified montmorillonite and vermiculite). Mineralogy and particle size did not differ significantly between treatments allowing the effect of contrasting land-use to be examined without confounding effects of mineralogy or particle size (Jensen et al., 2019).

Long-term ley-arable and bare fallow experiments at the site provided ‘previous’ grassland and arable (both since 1949, in 50 × 7 m plots in a randomized complete block design with four blocks) and ‘previous’ bare fallow (since 1959, in adjacent land totalling *c*. 1400 m^2^) land-uses, as described elsewhere (Gregory et al., 2016). The grassland management supports predominantly rye grass (*Lolium perenne* L.) and clover (*Trifolium* spp.), mown twice in summer with arable represented by winter wheat (*Triticum aestivum* L.) formerly in rotation with winter oats (*Avena sativa* L.). Hay and straw residues are removed after cutting. The fallow treatment is maintained by mouldboard ploughing and cultivating two to four times a year. Arable plots receive 220 kg N ha^−1^ annually (primarily as ammonium nitrate, with occasional ammonium sulphate to supply crop S). Selected properties of the long-term treatments are given in Table 1.

**Table 1:** Selected physical and chemical properties of the soil (upper 10 cm) at Highfield under long-term grass and arable (since 1949) and fallow (since 1959) land uses as measured in 2005 by Gregory et al. (2009) and Watts (personal communication) unless stated.

Property	Unit	Grass	Arable	Fallow
Sand	g kg^−1^	179	189	178
Silt	g kg^−1^	487	504	525
Clay	g kg^−1^	333	306	297
Particle density	g cm^−3^	2.46	2.57	2.61
Bulk density^a^	g cm^−3^	0.99	1.44	1.32
Plastic limit	g kg^−1^	484	225	208
Liquid limit	g kg^−1^	663	364	319
Plasticity index	%	17.9	13.9	11.1
Linear shrinkage	%	11.0	10.0	8.2
Water content –1 kPa	g kg^−1^	449	254	231
Water content –5 kPa	g kg^−1^	407	244	219
Water content –30 kPa	g kg^−1^	355	234	221
Water content –300 kPa	g kg^−1^	178	126	115
Water content –1500 kPa	g kg^−1^	176	115	99
Exchangeable Ca	mmol_c_ kg^−1^	61.8	23.8	10.3
Exchangeable Mg	mmol_c_ kg^−1^	6.0	3.5	4.2
Exchangeable Mn	mmol_c_ kg^−1^	11	9.1	4.2
Exchangeable K	mmol_c_ kg^−1^	4.2	1.4	0.8
Exchangeable Na	mmol_c_ kg^−1^	1.0	1.9	0.8
Cation exchange capacity	mmol_c_ kg^−1^	78.9	36.4	18.7
pH	–log (g [H^+^] L^–1^)	6.30	5.76	4.43
Electrical conductivity	mS cm^−1^	0.21	0.08	0.05

a Gregory et al. (2016).

In October 2008, three split-plots (10 × 6 m each) were established on each previous grassland and arable treatment in all four blocks, though in the present study we only focussed on the first three. Three sets of three split-plots (of the same size) were established on the adjacent fallow treatment to achieve the same replication. In the split-plots, the same three treatments (grassland, arable and fallow) were established as ‘current’ management treatments. Thus, the experimental design comprises 9 treatments triplicated in 27 split-plots (3 previous × 3 current management treatments × 3 field replicates). Each of the 9 treatments are therefore characterised by both a *previous* and a *current* management component. This temporal separation implicitly establishes a contrast in the abundance of management-driven transient binding agents (with transient agents being more abundant under the current managements which favour them). Hereafter we use the terms ‘grass’, ‘arable’ and ‘fallow’ to describe the management treatments and the symbol → to indicate where a previous management has been changed to a new current management. For example, grass → arable indicates the treatment previously under grass, but currently under arable.

### 2.2 Sampling and analysis

Soil samples were collected from three random locations in each of the 27 split-plots in February 2011 with a 75-mm diameter auger to a depth of 52 mm. To reduce within-plot variation as much as practicable, sub-samples were bulked to make a single sample per plot. A natural 3–5 mm aggregate fraction was teased from the bulk after partial-drying in a dark, 20 °C ventilated chamber for 18 h to prevent wet-smearing of aggregates upon handling. Roots and macrofauna were removed by hand. The majority of the 3–5 mm fraction was further air-dried in preparation for physical and chemical analyses while portions of the moist 3–5 mm aggregates were crumbled to pass through a 2 mm sieve and chilled to 4 °C overnight. EPS was then extracted as described by Redmile-Gordon et al. (2014). Accordingly, readily soluble organic material was removed from about 3.0 g moist soil (2.5 g dry weight equivalent of soil from each plot) using 0.01 M CaCl_2_ at a 1:10 soil:solution ratio, in 50 mL capacity polypropylene centrifuge tubes. These tubes were packed in ice and placed on an end-end reciprocating shaker set to 120 rev. min^−1^. Following this they were centrifuged at 3200 *g* for 30 min. Supernatant was discarded. EPS was then extracted from the remaining pellet. This was done by re-suspending the pellet in extraction buffer (2 mM Na_3_PO_4_·12H_2_O (0.760 g L^-1^), 4 mM NaH_2_PO_4_·H_2_O (0.552 g L^-1^), 9 mM NaCl (0.526 g L^-1^), 1 mM KCl (0.0746 g L^-1^), adjusted to pH 7 with 1 M HCl and pre-cooled to 4 °C. CER (Dowex ‘Marathon C’ sodium form, strongly acidic, 20–50 mesh) was prewashed twice in the above buffer, and 15.98 g CER was added together with the 25 mL buffer. This amount of CER was determined on the basis of 178 mg CER mg^−1^ SOC, as required by the soil with highest SOC content. Tubes were shaken for 2 h, and then centrifuged at 4000 *g* for 30 min. The supernatant containing EPS was transferred into new tubes, frozen in liquid N_2_, and stored at −80 °C prior to analysis. Total polysaccharide and uronic acids were quantified as described by Dubois et al. (1956) and Mojica et al. (2007), respectively. Extracted protein was measured using a Lowry based technique with reagent concentrations modified for microplate format as described by Redmile-Gordon et al. (2013), except that no additions of model polyphenolics or dilutions of soil extracts were required. As in previous work, EPS-protein was analysed by comparison to standards of bovine serum albumin (BSA; Sigma A7906) prepared in EPS buffer. Redmile-Gordon et al., (2013) tested the approach proposed by Frolund et al. (1995) to correct for interferences in the extracts. The interference of non-proteinaceous chromogens in natural soil extracts was well described. Accordingly, the interference from non-proteinaceous chromogens in the soil extracts was corrected for by i) measuring the absorbance of a second set of samples using Lowry reagents without CuSO_4_, and ii) applying the mathematical correction as presented by Frolund et al., (1995) i.e.

(1) $$\mathrm{Ab}s_{\mathrm{protein}}=\;1.25\left(\mathrm{Ab}s_{A}-\;Abs_{B}\right)$$

Where *Abs_A_* is the absorbance given for soil extracts using Lowry reagents, *Abs_B_* is the absorbance given by the set analysed using Lowry reagents without added CuSO_4_, and *Abs_protein_* is the theoretical absorbance due to protein, which is compared against a standard curve of BSA standards made in EPS extraction buffer.

SOC and total N concentrations were determined on the 3–5 mm fraction which was firstly air-dried, and fine-milled (<355 µm) before dry combustion in a Leco TruMac Combustion Analyser (LECO Corp., St Joseph, MI, USA) after carbonate removal with HCl. The MWD (µm) of stable 3–5 mm aggregates was obtained following the procedure for slaking by ‘fast wetting’ described by Le Bissonnais, 1996, ISO, 2012). We selected the ‘fast wetting’ component of the test as this assesses the response of soil to a highly-disaggregating force, as controlled by the strength of aggregates and their wetting rate.

### 2.3 Statistical analyses

All statistical analyses were completed using the GenStat (18th Edition) programme (VSN International Ltd., Hemel Hempstead, UK). We first assessed whether the residuals were normally distributed, and transformed (log_10_) the SOC, N and MWD variates accordingly. The complexity of the previous treatments in the Highfield experiments prior to the imposition of the current treatments was recently described by Gregory et al. (2016). Following their approach, we tested for significant effects of previous and current management treatments on the variates measured using analysis of variance (ANOVA) with the following model structures:

(2) $$\mathrm{treatment}:\left(\mathrm{experiment}/previous\right)*current$$

(3) $$\mathrm{block}:\left(\mathrm{experiment}\cdot \mathrm{block}\right)/plot/split-plot$$

The ‘previous’ treatment factor was first nested (/) within an ‘experiment’ factor which allocated data according to whether it derived from either the original ley-arable or the bare fallow experiment, prior to forming the cross-products (*) with the ‘current’ treatment factor. Likewise, the block model confirmed that there was no single block factor, but rather there were separate blocks for the different ‘experiments’ interacting (∙). By convention in a factorial design, we compared the effects of the ‘previous’ and ‘current’ land use main factors (treatments averaged over their current and previous land use, respectively; *n* = 9), and their interaction (*n* = 3). We deemed treatment differences to be ‘significant’ if the probability (p) of the ANOVA variance ratio statistic (F) was <0.05. We also conducted regression analysis to test for significant relationships between SOC, EPS and MWD variates.

## 3 Results

As an individual factor, previous land-use had no significant legacy effects on soil aggregate stability (MWD) or EPS (Table 2). However, there was a strong legacy effect on total SOC and N concentrations which decreased significantly in the order grass > arable > fallow (p < 0.05; Table 2). A significant effect was also observed for current management with SOC and N (p < 0.05) all increasing under grass with the exception that there was no significant difference ascribed to current management between the SOC concentrations currently under arable and fallow (Table 2). Moreover, the extractable EPS-protein, EPS-polysaccharide and EPS-uronic acid pools were all strongly affected by current management, with the variates being significantly greater under grass compared to either arable or fallow (p < 0.05), which did not differ significantly. Stable aggregate MWD was also only influenced by current management (Table 2), being significantly greater for soil under grass compared to arable, and significantly greater under arable compared to fallow (p < 0.05; Table 3). Under the scheme of Le Bissonnais (1996), soils currently under grass, arable and fallow are classed as ‘stable’, ‘unstable’ and ‘very unstable’, respectively.

**Table 2:** Mean ± standard error of the mean of soil organic C (SOC), total N (N), and soil extracellular polymeric substances (EPS) concentrations (protein, polysaccharide and uronic acid; µg EPS g^−1^ soil), and stable aggregate mean weight diameter (MWD), grouped by land uses (*n* = 3), and their interaction (*n* = 9). Where significant at *p* < 0.05, the least significant differences (LSD) of means are given (‘ns’ indicates non-significance). Note that some variates were transformed by log_10_ firstly to normalise the distribution of residuals.

Land use		SOC			N			EPS						MWD		
								protein		polysaccharide		uronic acid				
		(%)		(log_10_)%	(%)		(log_10_)%			(µg g^−1^)				(µm)		(log_10_)µm
*Previous*																
Grass		2.83	±0.27	0.439	0.26	±0.02	–0.598	188	±12	327	±16	144	±7	1157	±307	2.96
Arable		1.86	±0.13	0.262	0.18	±0.01	–0.753	186	±10	376	±24	203	±16	718	±124	2.81
Fallow		1.12	±0.09	0.038	0.11	±0.01	–0.974	143	±15	301	±27	180	±17	437	±52	2.61
*p*				0.017			0.035	0.823		0.285		0.086				0.137
LSD				0.100			0.129	ns		ns		ns				ns
*Current*																
Grass		2.49	±0.36	0.362	0.22	±0.03	–0.681	201	±11	387	±16	209	±16	1354	±276	3.06
Arable		1.73	±0.22	0.211	0.17	±0.02	–0.804	163	±16	298	±22	152	±11	573	±45	2.75
Fallow		1.59	±0.21	0.166	0.15	±0.02	–0.840	153	±11	319	±25	166	±15	384	±37	2.57
*p*				<0.001			<0.001	0.014		0.005		0.002				<0.001
LSD				0.053			0.052	31		50		28				0.07
*Previous*	*Current*															
Grass	Grass	3.67	±0.56	0.553	0.32	±0.05	–0.503	213	±21	346	±9	152	±4	2293	±388	3.35
	Arable	2.51	±0.19	0.397	0.23	±0.02	–0.632	195	±11	340	±24	152	±11	684	±43	2.83
	Fallow	2.33	±0.06	0.367	0.22	±0.01	–0.659	157	±20	294	±41	129	±19	494	±49	2.69
Arable	Grass	2.39	±0.09	0.377	0.22	±0.01	–0.653	198	±2	435	±13	248	±2	1174	±109	3.07
	Arable	1.64	±0.04	0.215	0.16	±0.00	–0.787	185	±31	318	±45	167	±34	584	±82	2.76
	Fallow	1.56	±0.03	0.194	0.15	±0.00	–0.818	176	±13	375	±37	194	±12	395	±27	2.60
Fallow	Grass	1.43	±0.05	0.155	0.13	±0.01	–0.888	191	±29	382	±31	227	±19	595	±61	2.77
	Arable	1.05	±0.06	0.021	0.10	±0.00	–0.992	111	±5	236	±3	136	±2	450	±42	2.65
	Fallow	0.87	±0.05	–0.063	0.09	±0.00	–1.041	126	±14	287	±46	177	±30	264	±1	2.42
*p*				0.988			0.988	0.597		0.133		0.073				0.035
LSD				ns			ns	ns		ns		ns				0.16

**Table 3:** Stable aggregate mean weight diameter ((log_10_) µm) as a linear function of soil organic C (SOC; %) and soil extracellular polymeric substances (EPS) concentrations (protein, polysaccharide and uronic acid; µg EPS g^−1^ soil). The table gives the constant (*a*) and coefficient (*b*) of the linear regression (*y* = *a* + *bx*), the probability level associated with the regression (*p*), and the adjusted proportion of the variance accounted for by the fit (Adjusted *R*_2_). Note that for uronic acid, the adjusted *R*_2_ is not calculable as the residual variance exceeded the variance of the response variate.

Statistic	SOC	EPS		
		protein	Polysaccharide	Uronic acid
Constant	2.29 ± 0.07	2.17 ± 0.18	2.26 ± 0.23	2.64 ± 0.21
Coefficient	0.26 ± 0.03	0.0036 ± 0.0010	0.0016 ± 0.0007	0.0008 ± 0.0011
*p*	<0.001	0.002	0.027	0.465
Adjusted *R*^2^	0.719	0.301	0.149	not calculable

The only variate that revealed a significant interaction between previous and current land-use was MWD (p = 0.035; Table 2). Within each previous management treatment, MWD showed statistically significant differences (p < 0.05) as a result of current land-use: MWD being greatest for grass, intermediate for arable, and smallest for fallow within just 2.5 years from establishing the current land-use. Plots previously under grass showed enhanced structural stability when the current land-use was also grass (grass → grass).

**Fig. 2:**
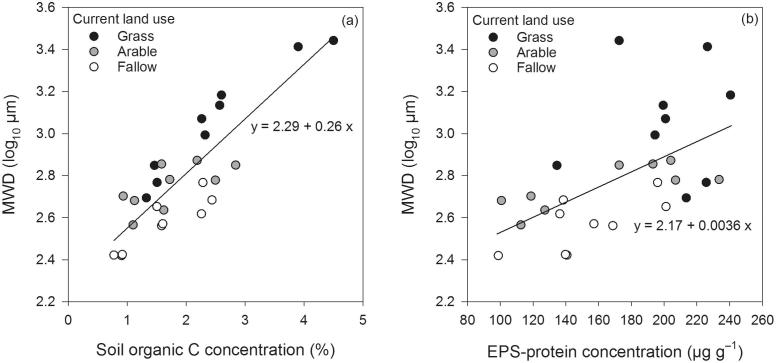
Stable aggregate mean weight diameter (MWD) as a linear function of (a) soil organic C concentration (y-axis), and (b) EPS-protein concentration. Current grass, arable and fallow land uses are shown (black, grey and white symbols, respectively). See Table 3 for further details of the linear regression analyses.

Significant regression models (p < 0.05) were found for MWD (log_10_) as a function of SOC, EPS-protein and EPS-polysaccharide (adjusted *R*^2^ = 0.72, 0.30, and 0.15, respectively; Table 3). Accordingly, these are presented as Fig. 2.

Notably, all soils under grass appeared to exhibit MWDs in excess of those predicted by the regression between MWD and SOC (Fig. 2). If it is assumed that the measured organic substances contribute to an increase in MWD (as is supposed; Tisdall and Oades, 1982) then the upper limit of the magnitude of the ‘binding effect’ per unit binder on a weight-for-weight basis (w/w) can be compared by standardising SOC units to the same scale as EPS parameters (Table 3). Standardising all to mg binder g^−1^ soil gives mass-adjusted coefficients for SOC, EPS-polysaccharide, and EPS-protein of 0.027, 1.6, and 3.6, respectively.

**Fig. 3:**
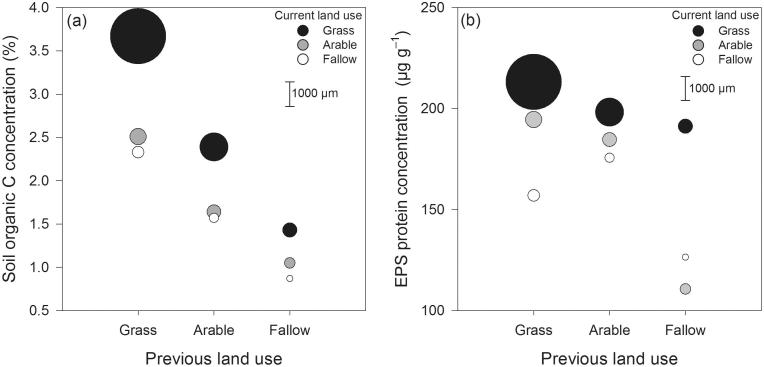
Mean (*n* = 3) (a) soil organic C concentration (y-axis), and (b) EPS-protein concentration as related to stable aggregate mean weight diameter (MWD; circles) for the nine previous × current land use combinations. Previous land use (x-axis) is shown in factorial combination with current grass, arable and fallow land uses (black, grey and white symbols, respectively). The diameter of each circle represents the stability (scale bar at 1000 µm MWD).

Since there was a significant previous–current land use interaction on MWD (Table 2) we explored the links to SOC and EPS-protein by plotting the two most significant predictors of MWD on the natural scale (Fig. 3). This provides visual clarity that something subtler than total SOC is affecting the MWD, and that appreciable changes in aggregate stability have been achieved within 2.5 years of the new managements. For example, soils previously under grass retained greater SOC contents, but the MWDs of these treatments (grass → arable and grass → fallow) were only about half those of the arable → grass soils. Furthermore, arable → grass soils contained less SOC than grass → arable (Fig. 3a). Accordingly, prediction of the expected stability order, based on ranking of SOC content was successful in only 3 out of 9 cases with a mismatch occurring between SOC rank and MWD order for most land-use combinations (Fig. 3a). In contrast, EPS-protein (Fig. 3b) enabled better prediction of rankings for management-driven stabilities across the range of stability classes from ‘very stable’ to ‘unstable’ (Fig. 3b): being successful in 6 out of 9 cases (Fig. 3b). This relationship was more apparent with the more stable soils, where rank of EPS-protein matched the order of the top five structural stabilities: greatest EPS-protein for the greatest MWD, second greatest EPS-protein for the second greatest MWD, and so on.

All treatments previously under grass showed the greatest changes in SOC, with losses outweighing the loss or gain of any other permutation. This was not the case with EPS-protein (Fig. 3b) where the magnitude of increase from fallow (fallow → grass) was similar to the loss of EPS-protein when transitioning from the reverse permutation (grass → fallow). There were greater losses of MWD, SOC and EPS-protein associated with transition to fallow (grass → fallow) over arable (grass → arable). Both transitions resulted in aggregates being classed as ‘unstable’ compared to those remaining under grass which were ‘very stable’ (Le Bissonnais, 1996). In contrast, soils previously under arable showed very little loss in SOC or EPS-protein content (1.64–1.56%; and 185–176 µg g^−1^) when converted to fallow, but the decrease in MWD was much greater: from 584 to 395 µm with a change of class from ‘unstable’ to ‘very unstable’ (Le Bissonnais, 1996).

The greatest total improvement in MWD was seen with transitions from arable to grass (increase of 590 µm), and while the SOC content of this treatment was less than the SOC content of the reverse grass → arable treatment, the EPS-protein was indeed greater, and the MWD was much greater too (at 1174 ‘medium’ *vs.* 684 µm ‘unstable’; Le Bissonnais, 1996). Likewise, comparing the grass → fallow with fallow → grass treatment changes we see that the former retained a considerably greater SOC concentration (2.33 *vs.* 1.43%) but a considerably lesser EPS-protein concentration (157 *vs.* 191 µg g^−1^), while the latter had the greater EPS-protein concentration and structural stability as indicated by MWD (494 *vs.* 595 µm; Table 2).

We established that there was no significant interaction effect between previous × current land use on EPS fractions, however we did note some unexpected observations. EPS-polysaccharide decreased with a transition from fallow to arable, as did EPS-protein (Table 2), but apart from these soils previously managed as fallow, there were no other similarities in land-use responses between these EPS pools. EPS-polysaccharide and EPS-uronic acid both unexpectedly increased when converted to the reduced C-input fallow (Table 2; arable → fallow; 318 to 375 µg EPS-polysaccharide; 167 to 194 µg EPS-uronic acid g^−1^ soil). Accordingly, the reverse fallow → arable conversion decreased all EPS fractions, but increased the MWD (Table 2). Additionally, the greatest MWD of all land-uses (grass → grass) was associated with merely average EPS-polysaccharide concentrations.

## 4 Discussion

### 4.1 Effects of land-use and land-use change on SOC, EPS and MWD

Continuous grassland clearly resulted in the greatest SOC, EPS and structural stability (MWD). We found total SOC concentration to be the best *overall* predictor of MWD according to the adjusted R^2^ (Table 3). However, the maximum possible contribution of SOC to MWD was relatively low when considered on an equivalent mass basis: about 60 or 140× smaller than that of EPS-polysaccharide or EPS-protein, respectively (Table 3). This stands to reason as not all SOC will have a binding effect. This also suggests that the relationship may not always be sensitive enough to describe how small changes in SOC affect a soil’s stability. The lack of a decisive relationship between SOC and aggregate stability has been identified before (Tisdall and Oades, 1982, Liu et al., 2005, Denef et al., 2007, Sarker et al., 2018) and we explore this further in our data.

We found greater scattering in EPS-protein’s relationship to MWD from the data shown in Fig. 2b compared to Fig. 2a, which could be due to variability introduced during the extraction of EPS prior to measurement, incomplete extraction, and/or reflect some natural underlying variability in the relationship. Indeed, one of the strengths of the total SOC measurement is the complete combustion of C in the analysis. Nonetheless, comparisons of Fig. 3a and b show that EPS-protein may be more mechanistic in the formation —or maintenance— of the more stable aggregates in response to land-use and land-use change. For example, total SOC (Fig. 3a) did not explain the mean stability order of land-use combinations, with a mismatch occurring between SOC rank and MWD rank in most cases. In contrast, EPS-protein, which was controlled by current land-use (Table 1), described the stability order more accurately (Fig. 3b). From a biofilm perspective this might be expected, because proteinaceous moieties in EPS are widely known to mediate biological attachment to abiotic surfaces (e.g. Taglialegna et al., 2016). A notable example of these impacts on stability at odds with total SOC is found in the extreme land-use changes between grass and fallow. While grass → fallow retained considerably greater SOC than fallow → grass (2.33 *vs.* 1.43%), the latter yielded more EPS-protein (191 *vs*. 157 µg g^−1^) and with this, significantly greater structural stability (MWD; 595 *vs*. 494 µm; Table 2).

There were examples where considerable decreases in MWD were not fully explained by either SOC or EPS. Treatments previously under arable showed very little loss in SOC when converted to fallow (1.64–1.56%, a relative decrease of 5%), but the decline in soil physical stability (MWD) was much greater and significant (p < 0.05) from 584 µm to 395 µm: a relative decrease of 32%, or transition from qualitative classes ‘unstable’ to ‘very unstable’ (Le Bissonnais, 1996). In this instance, the loss of EPS-protein was similar to SOC (185–176 µg EPS g^−1^; relative decrease of 5%). This decline is likely to be more related to differences in *temporary* binding agents (plant roots and hyphae) because increased tillage is well-known known for its disruptive influence on fungal hyphae in fallow soils (Kabir, 2005). This magnitude of decline in aggregate stability is also similar to that found by Blankinship et al. (2016) where plants were manually removed from a seasonally dry grassland. We also found examples where a considerable *increase* in MWD was not fully explained by either SOC or EPS. The transition from arable to grass corresponded to increases of 7–48% in the EPS fractions and a 46% increase in SOC but a much greater (101%) increase in MWD. As above this suggests temporary binding agents were also implicated. Grass leys in arable rotation are widely recognised for their capacity to improve soil physical quality, which is understood to be largely owed to improvements in both temporary and transient binding agents (Tisdall and Oades, 1982). Accordingly, the size of the microbial biomass and abundance of fungi at our experimental site were reported by other authors to increase in the order fallow > arable > grass (Gregory et al., 2009; Hirsch et al., 2009; Wu et al., 2012). In the present study, the only binding agents measured were *transient*. However, most roots were removed in the preparation (Section 2.2) and so we assume that fungal hyphae likely accounted for the majority of influence from temporary binding agents.

Considering the positive interaction between previous and current land-uses observed for MWD (p < 0.05; Table 2; Fig. 3a) it is apparent that the beneficial effects of current grass and arable land-uses on MWD must have been enhanced by specific conditions at the time of land-use change (owed to the preceding 50 year’s land-use). Root establishment of the grass ley and winter wheat (2.5 years prior to sampling) would have clearly benefitted from the soil physical and chemical properties originating from the previous grass land-use (Table 1). Increased plant cover and/or reduced tillage therefore can be seen to have provided positive feedback for the subsequently beneficial land-uses. Nevertheless, no measurable legacy effects or interactions were detected for any of the extracted EPS (Table 2) confirming that the EPS themselves were *transient.* Furthermore, it is not likely that *persistent* binding agents declined substantially in the 2.5 years following land-use change, because organo-mineral associations tend to be resistant to oxidation and microbial metabolism (Kögel-Knabner et al., 2008). Therefore, we expect that this interaction effect is due to *temporary* binding agents co-existing at the time of sampling. Both temporary and transient binding agents are predominantly composed of relatively ‘labile’ carbon compounds, which are a temporally unstable pool known to decline relative to total SOC in response to reduced plant cover and increased tillage (Haynes, 2000).

These combined findings above support the claim of Jensen et al. (2019) that distinct components of SOC, rather than size of the total pool, have a disproportionate effect on aggregate stability. We also observed a larger range, and greater maximum total SOC between previous land-use classes than between current ones (Table 2). This suggests that total SOC content was primarily owed to previous land-use, and moreover that the bulk of SOC in soil previously under grass persisted >2.5 years. This is supported by historically fallow plots containing the least SOC whereas historically grass-covered plots contain the most (Table 2). While the concentration of ‘persistent’ or ‘older’ SOC cannot be used as a surrogate for *persistent binding agents*, it is important that this large quantity of organic material associated with previous land-uses does not appear to contribute significantly to the stability of soils in our study (see MWD; Table 2; p = 0.137). Furthermore, this form —or location— of SOC was not readily available for production of microbial EPS.

In accordance with the present findings, Redmile-Gordon et al. (2015) took soils from the same grass → arable plots in February 2012 and found that this large pool of SOC did not support microbial assimilation of inorganic N (^15^NH_4_^15^NO_3_) provided during incubation in the laboratory. Neither was there any exudation of ^15^N labelled EPS into the extracellular space *unless* ^15^N was accompanied with simultaneous inputs of labile C. The above study highlighted the importance of fresh C inputs for EPS production, which is reflected in the present findings of elevated quantities of EPS-protein, EPS-polysaccharide and EPS-uronic acids in soils under dense vegetation cover at the time of sampling (i.e. grass; Table 2). Moreover, these findings reinforce the notion of a ‘turning point’ where older SOC, which is presumably more decomposed or physically occluded, ceases to be a viable source of C to measurably support exudation of EPS. This is further illustrated by the considerably greater EPS concentrations (protein, polysaccharide and uronic acid) under fallow → grass compared to grass → fallow (Table 2) and thus supports the theory presented by Golchin et al. (1994). Our findings show that measurement of ‘transient’ binding agents using the method of Redmile-Gordon et al., (2014) can add useful depth and contrast to measures of total SOC.

### 4.2 SOC *vs*. biological binding agents as drivers of soil stability

There remains a small paradox to be settled in that SOC shows a good correlation to aggregate stability, while the previous land-managements that contribute the majority of this SOC were not directly related to the MWD observed (Table 2; p = 0.137). However, as discussed in section 4.1, a positive interaction arising from the previous grass land-use was driving improved soil quality generally, for example with greater SOC contributing a much reduced bulk density (Table 1). More successful plant establishment in 2008 would therefore be expected, and is confirmed by yield measurements taken by other investigators at the same site (e.g. Hirsch et al., 2017). This increased plant biomass would in turn have increased the abundance of associated binding agents such as roots and hyphae – contributing to the MWD measured at the site. Importantly, this clarifies an important point that residual SOC needs no *direct* contribution to soil stability to indirectly contribute to a good R^2^ for MWD via positive feedback effects. This example illustrates the case that large quantities of ‘inactive’ SOC can contribute relatively small concentrations of temporary and transient binding agents, which are overwhelmed by the contributions from current land-use (Table 2; Fig. 3b).

While it could be argued that EPS production may also have been boosted by the same factors presented in Table 1, this fraction is sufficiently ‘transient’ that any increases in EPS that occurred previous to the moment of land-use change were no longer detectable at the time of sampling (Table 2). However, there would have been an abundance of EPS in plots formerly under grass, which interestingly corresponds to the increase in exchangeable multivalent cations (Ca^2+^ and Mg^2+^) under this land-use (Table 1). As discussed in Section 4.1, fresh C was previously seen to be vital for production of EPS in these soils. Multivalent cations not only bridge carboxylic acid moieties within the EPS, but when sufficiently concentrated, contribute electrostatic interactions between the EPS and negatively charged mineral surfaces to form considerably stable bonds (Berne et al., 2015). We propose that electrostatic bonding between clays and biota could therefore depend on soil biota being able to maintain production of EPS to retain anchorage of these cations. Our data suggest that the majority of all stabilising effects subsided within 2.5 years of transition away from the greater-C grass land-use, with MWD of grass → arable becoming similar to that of continuous arable by the time of measurement (Table 2; LSD 0.16 log_10_ µm).

### 4.3 EPS-protein *vs*. EPS-polysaccharide

While Tisdall and Oades (1982) claimed that extracellular polysaccharides would be the most important transient binding agents in soil, investigations of biofilms in other environments show that protein and polysaccharide pools are not mutually exclusive, with glycoproteins typically being abundant, and essential, in bonding (Flemming et al., 2007). Among the transient EPS parameters measured here, EPS-protein showed the greatest coefficient of regression and R^2^ against MWD (Table 3). Proteinaceous moieties in the EPS matrix are especially recognised for their contribution to stable biofilm structure (Vlamakis et al., 2013). The relatively low R^2^ for polysaccharide and protein (0.15, and 0.30, respectively) are expected because the contributions of *temporary* (and *persistent*) binding agents are, of course, excluded.

With regard to the mechanisms underlying the apparently EPS-protein driven stabilisation in the more stable soils (Fig. 3b), BslA proteins exuded from the model soil organism (*Bacillus subtilis*) were found to self-assemble extracellularly: providing architectural stability and protecting the otherwise fragile polysaccharides against disruption from fast wetting (Arnaouteli et al., 2016). Earlier work found that hydrophobic amino acids —most notably phenylalanine and tyrosine— were characteristic of community EPS produced in response to a hydrophilic source of C (Redmile-Gordon et al., 2015). Tyrosine has since been shown to be of mechanistic importance in the formation of adhesive peptides, which impart strength and elasticity to ‘living glues’ produced by soil organisms (Zhang et al., 2019). The elevated concentrations of proteinaceous EPS in soils currently under grass (Table 2) is perhaps not surprising given that increased availability of plant polysaccharides is known to trigger production of extracellular proteins in the laboratory (Beauregard et al., 2013). However, given that in the present study a) these pools of EPS are transient, and b) solar radiation was at a seasonal low, it is somewhat surprising that these increases in community EPS were still measurable in the field by extraction in the temperate midwinter. Whether this is indicative of basal winter photosynthesis supporting sufficient rhizodeposit-C or a minimum turnover rate equal to several months we cannot say. Nonetheless, the majority do not remain extractable for more than 2.5 years, with previous land-use having no direct effect on the size of this pool (p = 0.823; Table 2).

Exceptions to the straightforward relationship of [increased plant-C] + [decreased tillage] = [greater EPS] are seen with land-use changes between fallow and arable. Fallow → arable conversion, with its implicit increases in C-inputs and decreased tillage, showed marked declines in EPS-polysaccharide and uronic acid fractions amounting to −51 and −41 µg EPS g^−1^ soil, respectively (Table 2). Likewise, arable → fallow conversion had notably increased EPS-polysaccharide and EPS-uronic acid fractions compared to its arable → arable counterpart, despite clearly greater C inputs from winter wheat and comparatively reduced tillage. Precedent exists for two feasible explanations. Firstly, the increased solar radiation for cyanobacteria and other photoautotrophs on the fallow surface may enable sufficient production of extracellular polysaccharides to be detected in the bulk soil. Cyanobacterial polysaccharides can stabilise soil surface crusts (Cania et al., 2019) but are apparently not highly influential for the stability of bulk soil (Table 2). Secondly, and it seems more likely, the inorganic N fertiliser applied annually to these arable plots somehow reduced the efficiency of microbial EPS production in the bulk soil (mass of EPS per unit microbial ATP), as shown previously by Redmile-Gordon et al. (2015). In every other sense, the arable land-use represents the ‘intermediate’ of individual factors contributing to the contrasting land-use pressures, i.e. tillage frequency, C inputs, duration of vegetative cover, and pH (Table 1). Wu et al. (2012) also reported intermediate soil microbial biomass concentrations, as expected between grass and fallow. Nonetheless, the arable land-use is exceptional in that inorganic N fertiliser is applied annually. Our data therefore support the conceptual model proposed by Redmile-Gordon et al. (2015) that while C is a fundamental requirement for significant EPS production, surplus inorganic N acts as a ‘switch’: shifting soil microbial investment of C from EPS to intracellular investment for growth of cells.

### 4.4 Implications for land-use and soil management strategies

Most SOC (>98%) in sieved soil taken from a grass → arable plot of the same long-term experiment was shown to be non-living and inaccessible to the soil microbial biomass (Redmile-Gordon et al., 2015). In the present study, the residual fraction of SOC from the same previous land-use was also found to not be directly influential for soil structural stability (Table 2) with the primary binding agents operating at our study site being attributed to the influence of current land-use over the long-term previous land-use (Table 2). Taken together, these results suggest that soil biology, or biological binding agents are vital for favourable soil quality (MWD). The significant shifts in this reasonably ‘holistic’ measure of soil quality (Six et al., 2000) were achieved within a relatively short period (<2.5 years). Management of biological binding agents, including EPS as a highly responsive *transient binding agent* may therefore be useful to rapidly improve soil structural stability to meet various environmental objectives. The approach of building a smaller more ‘active’ subset of total SOC may also help to avoid some of the seasonal risks associated with large pools of total SOC, including emissions of carbon dioxide, leaching of N overwinter, contamination of rivers, aquifers, and the subsequent indirect emissions of nitrous oxide arising from leached N (Reay et al., 2012).

## 5 Conclusion

We confirmed that the primary binding agents affected by current land-use were transient and/or temporary. Direct effects of current mixed grass and clover, without fertiliser inputs, were more important for both soil physical stability, and EPS, than any legacy from a previous land-use (including greater SOC). Among the extracellular fractions studied, the stability of aggregates was most strongly related to increases in EPS-protein, less related to EPS-polysaccharide, and not related to uronic acids. We also demonstrated that measurement of transient binding agents, using a recently developed method, adds useful depth and contrast to measures of total SOC. We conclude that there may be significant environmental benefits to managing smaller specific ‘functional’ pools of C in agriculture and horticulture, rather than increasing total SOC *per se*.

## Declaration of Competing Interest

The authors declare that they have no known competing financial interests or personal relationships that could have appeared to influence the work reported in this paper.
