# Supplementary material for: Soil organic carbon, extracellular polymeric substances (EPS), and soil structural stability as affected by previous and current land-use
Source: Geoderma. 2020 Apr 1;363:114143. doi: 10.1016/j.geoderma.2019.114143 (PMC7043399; doi:10.1016/j.geoderma.2019.114143)
Supplement: Supplementary data 1 [file mmc1.docx]

**Supplementary Material**

**Table S1**

Full dataset of soil organic C (SOC), total N (N) and extracellular polymeric substances (EPS) concentrations (protein, polysaccharide and uronic acid), and stable aggregate mean weight diameter (MWD) variates under previous and current land uses and their interaction.

| Land use | | Block | Plot | Split-plot | SOC | N | EPS | | | MWD |
| --- | --- | --- | --- | --- | --- | --- | --- | --- | --- | --- |
| Previous | Current |  |  |  |  |  | protein | polysaccharide | uronic acid |  |
|  |  |  |  |  | (%) | | (µg g^–1^) | | | (µm) |
| Grass | Grass | 1 | 4 | 10 | 4.500 | 0.386 | 172.54 | 338.84 | 145.46 | 2768.57 |
| Grass | Grass | 2 | 6 | 17 | 3.900 | 0.349 | 226.46 | 364.06 | 151.83 | 2587.72 |
| Grass | Grass | 3 | 9 | 26 | 2.600 | 0.230 | 240.50 | 335.17 | 157.66 | 1524.06 |
|  |  |  |  | *Mean* | *3.667* | *0.322* | *213.17* | *346.02* | *151.65* | *2293.45* |
| Grass | Arable | 1 | 4 | 12 | 2.186 | 0.204 | 204.10 | 366.75 | 158.35 | 744.61 |
| Grass | Arable | 2 | 6 | 16 | 2.840 | 0.266 | 172.58 | 292.39 | 130.74 | 707.66 |
| Grass | Arable | 3 | 9 | 27 | 2.497 | 0.234 | 207.00 | 359.51 | 165.75 | 599.74 |
|  |  |  |  | *Mean* | *2.508* | *0.235* | *194.56* | *339.55* | *151.61* | *684.00* |
| Grass | Fallow | 1 | 4 | 11 | 2.285 | 0.211 | 196.00 | 366.98 | 163.26 | 584.45 |
| Grass | Fallow | 2 | 6 | 18 | 2.439 | 0.230 | 138.51 | 290.60 | 124.38 | 482.65 |
| Grass | Fallow | 3 | 9 | 25 | 2.260 | 0.217 | 136.36 | 224.40 | 98.01 | 414.90 |
|  |  |  |  | *Mean* | *2.328* | *0.219* | *156.96* | *293.99* | *128.55* | *494.00* |
| Arable | Grass | 1 | 5 | 13 | 2.570 | 0.240 | 199.42 | 455.70 | 244.38 | 1362.67 |
| Arable | Grass | 2 | 7 | 21 | 2.264 | 0.211 | 200.85 | 438.84 | 246.90 | 1175.54 |
| Arable | Grass | 3 | 8 | 23 | 2.323 | 0.217 | 194.46 | 410.06 | 251.60 | 983.72 |
|  |  |  |  | *Mean* | *2.386* | *0.223* | *198.24* | *434.87* | *247.63* | *1173.98* |
| Arable | Arable | 1 | 5 | 14 | 1.723 | 0.166 | 233.58 | 291.52 | 140.03 | 603.67 |
| Arable | Arable | 2 | 7 | 20 | 1.621 | 0.155 | 127.19 | 256.28 | 126.29 | 432.25 |
| Arable | Arable | 3 | 8 | 24 | 1.584 | 0.169 | 193.15 | 405.56 | 235.28 | 715.86 |
|  |  |  |  | *Mean* | *1.643* | *0.163* | *184.64* | *317.79* | *167.20* | *583.93* |
| Arable | Fallow | 1 | 5 | 15 | 1.585 | 0.154 | 168.71 | 325.95 | 174.81 | 364.27 |
| Arable | Fallow | 2 | 7 | 19 | 1.604 | 0.152 | 157.13 | 350.64 | 190.59 | 371.89 |
| Arable | Fallow | 3 | 8 | 22 | 1.505 | 0.150 | 201.16 | 447.40 | 216.15 | 449.17 |
|  |  |  |  | *Mean* | *1.565* | *0.152* | *175.67* | *374.66* | *193.85* | *395.11* |
| Fallow | Grass | 1 | 1 | 2 | 1.463 | 0.128 | 134.58 | 331.83 | 209.57 | 705.15 |
| Fallow | Grass | 2 | 2 | 6 | 1.507 | 0.141 | 225.71 | 437.72 | 264.38 | 585.13 |
| Fallow | Grass | 3 | 3 | 9 | 1.326 | 0.120 | 213.51 | 374.96 | 206.77 | 494.25 |
|  |  |  |  | *Mean* | *1.432* | *0.130* | *191.27* | *381.50* | *226.91* | *594.84* |
| Fallow | Arable | 1 | 1 | 1 | 1.125 | 0.108 | 100.68 | 229.42 | 139.56 | 479.84 |
| Fallow | Arable | 2 | 2 | 5 | 0.934 | 0.097 | 118.74 | 237.02 | 135.56 | 503.87 |
| Fallow | Arable | 3 | 3 | 7 | 1.101 | 0.101 | 112.59 | 240.20 | 133.63 | 367.60 |
|  |  |  |  | *Mean* | *1.053* | *0.102* | *110.67* | *235.55* | *136.25* | *450.44* |
| Fallow | Fallow | 1 | 1 | 3 | 0.908 | 0.094 | 98.82 | 195.51 | 124.95 | 262.58 |
| Fallow | Fallow | 2 | 2 | 4 | 0.777 | 0.084 | 140.71 | 335.48 | 229.02 | 263.91 |
| Fallow | Fallow | 3 | 3 | 8 | 0.919 | 0.095 | 139.76 | 329.90 | 175.98 | 265.82 |
|  |  |  |  | *Mean* | *0.868* | *0.091* | *126.43* | *286.96* | *176.65* | *264.10* |

**Table S2**

The analysis of variance (ANOVA) table for the soil organic C (SOC), total N (N) and extracellular polymeric substances (EPS) concentrations (protein, polysaccharide and uronic acid), and stable aggregate mean weight diameter (MWD) variates with the structures outlined in equations 1-2, showing the effect of previous (P) and current (C) land uses and their interaction. The table gives the degrees of freedom (df) associated with the factor (first number) and its residual (comma separated), together with the variance ratio statistic (*F*) and its probability level (*p*). Where significant at *p*<0.05, the standard error of differences (SED) and the least significant differences (LSD) of means are given. Note that some variates were firstly transformed by log_10_ to normalise the distribution of residuals.

| Variate | Unit | Factor | df | *F* | *p* | SED | LSD |
| --- | --- | --- | --- | --- | --- | --- | --- |
| SOC | (log_10_)% | P | 1,2 | 57.42 | 0.017 | 0.023 | 0.100 |
|  |  | C | 2,12 | 35.47 | <0.001 | 0.024 | 0.053 |
|  |  | P × C | 2,12 | 0.01 | 0.988 |  |  |
| N | (log_10_)% | P | 1,2 | 26.76 | 0.035 | 0.030 | 0.129 |
|  |  | C | 2,12 | 24.66 | <0.001 | 0.024 | 0.052 |
|  |  | P × C | 2,12 | 0.01 | 0.988 |  |  |
| EPS- protein | µg g^–1^ | P | 1,2 | 0.06 | 0.823 |  |  |
|  |  | C | 2,12 | 6.23 | 0.014 | 14.3 | 31.1 |
|  |  | P × C | 2,12 | 0.54 | 0.597 |  |  |
| EPS- polysaccharide | µg g^–1^ | P | 1,2 | 2.09 | 0.285 |  |  |
|  |  | C | 2,12 | 8.38 | 0.005 | 23.0 | 50.0 |
|  |  | P × C | 2,12 | 2.40 | 0.133 |  |  |
| EPS- uronic acid | µg g^–1^ | P | 1,2 | 10.12 | 0.086 |  |  |
|  |  | C | 2,12 | 10.51 | 0.002 | 12.9 | 28.2 |
|  |  | P × C | 2,12 | 3.29 | 0.073 |  |  |
| MWD | (log_10_)µm | P | 1,2 | 5.81 | 0.137 |  |  |
|  |  | C | 2,12 | 131.49 | <0.001 | 0.031 | 0.067 |
|  |  | P × C | 2,12 | 4.50 | 0.035 | 0.069 | 0.155 |

**Table S3**

Regression statistics of stable aggregate mean weight diameter ((log_10_) µm) as a linear function of soil organic C (SOC; %) and soil extracellular polymeric substances (EPS) concentrations (protein, polysaccharide and uronic acid; µg EPS g^–1^ soil). The table gives the constant (*a*) and coefficient (*b*) of the linear regression (*y* = *a* + *bx*), the variance ratio statistic (*F*), the probability level associated with the regression (*p*), the degrees of freedom (df) and mean square error (MSE) of the residual, and the adjusted proportion of the variance accounted for by the fit (Adjusted *R*_2_). Note that for uronic acid, the adjusted *R*_2_ is not calculable as the residual variance exceeded the variance of the response variate.

| Statistic | SOC | EPS | | |
| --- | --- | --- | --- | --- |
|  |  | protein | polysaccharide | uronic acid |
| Constant | 2.29±0.07 | 2.17±0.18 | 2.26±0.23 | 2.64±0.21 |
| Coefficient | 0.26±0.03 | 0.0036±0.0010 | 0.0016±0.0007 | 0.0008±0.0011 |
| *F* | 67.49 | 12.21 | 5.56 | 0.55 |
| *p* | <0.001 | 0.002 | 0.027 | 0.465 |
| df | 25 | 25 | 25 | 25 |
| MSE | 0.020 | 0.051 | 0.062 | 0.074 |
| Adjusted *R*^2^ | 0.719 | 0.301 | 0.149 | not calculable |
